# Developing and testing a prediction model for periodontal disease using machine learning and big electronic dental record data

**DOI:** 10.3389/frai.2022.979525

**Published:** 2022-10-13

**Authors:** Jay S. Patel, Chang Su, Marisol Tellez, Jasim M. Albandar, Rishi Rao, Vishnu Iyer, Evan Shi, Huanmei Wu

**Affiliations:** ^1^Health Informatics, Department of Health Services Administrations and Policy, College of Public Health, Temple University, Philadelphia, PA, United States; ^2^Department of Oral Health Sciences, Kornberg School of Dentistry, Temple University, Philadelphia, PA, United States; ^3^Department of Periodontology and Oral Implantology, Kornberg School of Dentistry, Temple University, Pennsylvania, PA, United States

**Keywords:** dental informatics, prediction model, electronic dental record, electronic health record, XGBoost, machine learning, periodontal disease, social determinants of health

## Abstract

Despite advances in periodontal disease (PD) research and periodontal treatments, 42% of the US population suffer from periodontitis. PD can be prevented if high-risk patients are identified early to provide preventive care. Prediction models can help assess risk for PD before initiation and progression; nevertheless, utilization of existing PD prediction models is seldom because of their suboptimal performance. This study aims to develop and test the PD prediction model using machine learning (ML) and electronic dental record (EDR) data that could provide large sample sizes and up-to-date information. A cohort of 27,138 dental patients and grouped PD diagnoses into: healthy control, mild PD, and severe PD was generated. The ML model (XGBoost) was trained (80% training data) and tested (20% testing data) with a total of 74 features extracted from the EDR. We used a five-fold cross-validation strategy to identify the optimal hyperparameters of the model for this one-*vs*.-all multi-class classification task. Our prediction model differentiated healthy patients *vs*. mild PD cases and mild PD *vs*. severe PD cases with an average area under the curve of 0.72. New associations and features compared to existing models were identified that include patient-level factors such as patient anxiety, chewing problems, speaking trouble, teeth grinding, alcohol consumption, injury to teeth, presence of removable partial dentures, self-image, recreational drugs (Heroin and Marijuana), medications affecting periodontium, and medical conditions such as osteoporosis, cancer, neurological conditions, infectious diseases, endocrine conditions, cardiovascular diseases, and gastroenterology conditions. This pilot study demonstrated promising results in predicting the risk of PD using ML and EDR data. The model may provide new information to the clinicians about the PD risks and the factors responsible for the disease progression to take preventive approaches. Further studies are warned to evaluate the prediction model's performance on the external dataset and determine its usability in clinical settings.

## Introduction

Despite advances in periodontal disease (PD) research and periodontal treatments, 42% of the US population have PD, which has led to tooth loss, poor quality of life, and increased healthcare cost (Eke et al., 2016, 2018). To date, limited studies show the effectiveness of current periodontal treatments in preventing disease progression and tooth loss based on patient characteristics (Pihlstrom et al., 1983; Farooqi et al., 2015). A major barrier is the difficulty of conducting randomized controlled trials with adequate numbers of patients over a long time because of several reasons, such as ethical reasons, expenses, and difficulty in enrollment and retaining patients for a longer time (Song et al., 2013; Thyvalikakath et al., 2020). Moreover, it is also well-studied that the PD can be prevented if the risk factors responsible for PD progression could be controlled by assessing patients' disease risk (Grossi et al., 1995; Genco and Borgnakke, 2013; Garcia et al., 2016). As a result, prediction models to assess patient-specific disease risk have been developed (Lang and Tonetti, 2003; Page et al., 2004; Chandra, 2007; Trombelli et al., 2009, 2017; Koshi et al., 2012; Meyer-Bäumer et al., 2012; Lang et al., 2015; Shimpi et al., 2019). However, the use of these tools in dental clinics is limited (Sai Sujai et al., 2015; Thyvalikakath et al., 2018) due to their suboptimal performance.

Researchers worldwide have developed risk assessment and predictions models to assess the risk of periodontitis (Lang and Tonetti, 2003; Persson et al., 2003; Page et al., 2004; Chandra, 2007; Trombelli et al., 2009; Meyer-Bäumer et al., 2012; Lang et al., 2015; Mullins et al., 2016; Shimpi et al., 2019). Typically, these risk assessment tools provide patients' disease risk into low, moderate, or high risks. Studies have demonstrated that these models have helped improve the documentation of patient-specific periodontal information. Moreover, some risk assessment models also provide evidence-based treatment recommendations, which eliminates the use of paper-based treatment recommendations guidelines. Despite these advances and advantages of using these tools for patient care, the use of these tools in clinics is seldom because for several reasons (Meyer-Bäumer et al., 2012; Alonso et al., 2013; Dhulipalla et al., 2015; Sai Sujai et al., 2015; Petersson et al., 2016). The existing tools are (1) not providing new information to the clinicians that they do not know, (2) not providing patient-specific disease risk because the same patient's risk scores were significantly different by different tools, and (3) the development of the tools are based on the evidence generated years or decades ago that may not represent the current patient population.

The increased availability of longitudinal patient care data electronically through the electronic dental record (EDR) offers an opportunity to characterize the present patient population's demographics, disease profiles to develop prediction models with up-to-date information (Song et al., 2013; Patel et al., 2018; Thyvalikakath et al., 2020). Moreover, advanced machine learning (ML) methods provide us with an opportunity to develop sophisticated data-driven models for PD. In medicine, ample studies have demonstrated the use of big EHR data and advanced ML methods to predict the risk for diseases. For instance, Simon et al. (2018) developed a prediction model for suicide attempts and suicide deaths using electronic health record (EHR) data. The authors found the prediction of suicide attempt and suicide death were 0.851 (C-statistics) and 0.861, respectively (Simon et al., 2018). Similarly, Tomašev et al. (2019) developed a prediction model to predict future acute kidney injury and found promising results. Their model predicts 55.8% of all inpatient episodes of acute kidney injury and 90.2% of all acute kidney injuries requiring subsequent dialysis administration (Tomašev et al., 2019).

Nevertheless, in dentistry, very few studies have utilized EDR data to develop a prediction model to predict the risk of periodontitis. Thyvalikakath Thankam et al. (2015), Hegde et al. (2019), and Shimpi et al. (2019) have utilized EDR data to predict the risk of periodontitis. These studies provided promising results; however, the lack of involvement of patients' social determinants of health and systemic health risk factors restricted the model's use. Moreover, periodontal findings such as bone loss and deeper periodontal pockets are apparent risk factors that clinicians already know. Clinicians would be rather interested to know the driving risk factors for periodontitis so that they can take preventive approaches.

Therefore, the objective of this study was to develop a data-driven prediction model for PD using an advanced ML model, XGBoost, to identify novel risk factors for prediction. In this data-driven model, the ML model decided the feature of importance rather than pre-assigning risk factors based on the experts' opinions or literature like in other studies. The results of this study would (1) determine the feasibility of using the big EDR and ML model to predict the risk of PD, (2) find new associations between systemic diseases, social and behavioral factors, and PD, and (3) provide pilot data to build a clinical decision support system to predict the risk of PD in a clinical setting.

## Methods

**Figure 1:**
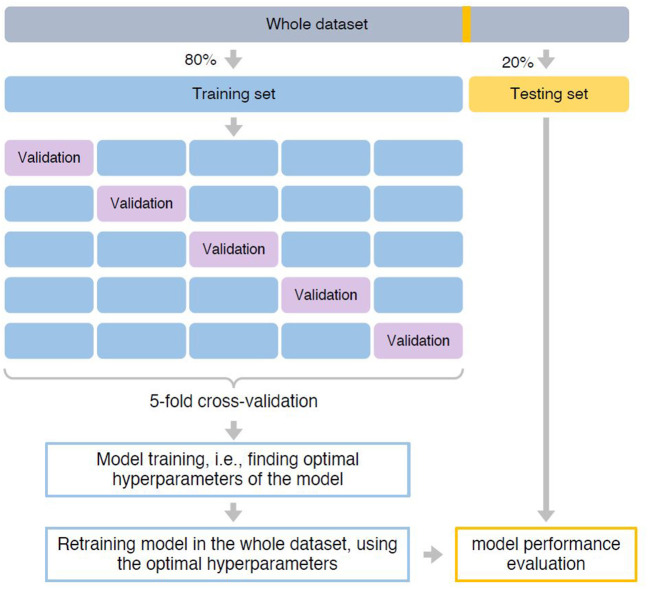
Pipeline of building the predictive model. The whole dataset was first split into 80% training and 20% testing set. The training set was used for model training, i.e., finding optimal hyperparameters that can achieve best prediction performance. We used a five-fold cross-validation strategy. Specifically, the training set was split into five-folds with equal size. Each time, a fold was used as the hold-out validation set to calculate model performance, while the remaining four-folds were used to train model. The procedure was repeated five times to make sure each fold was used as the hold-out validation set once. After identified optimal hyperparameters based on which the model can achieve the best prediction performance, we retrained the predictive mode using the whole training set. Finally, model performance was evaluated in the testing set.

This study was reviewed and approved by our institutional review board (Temple University Institutional Review Board Number: 28,321). In this retrospective study, the EDR data of patients who received at least one comprehensive oral examination (COE) in the Temple University Kornberg School of Dentistry (TUKSoD) predoctoral clinics were used. A cohort of 27,138 dental patients was generated and their demographics, medical history, periodontal findings, treatment information, and other 70 variables from the big EDR data were retrieved. The automated approaches to phenotype PD diagnoses and to retrieve patients' medical histories, social determinants of health, and behavioral habits from the free-text EDR data were generated. Last, a ML model was applied to predict the risk of periodontitis using more than 74 variables. The overall workflow of our methods is presented in Figure 1.

### Study cohort

**Figure 2:**
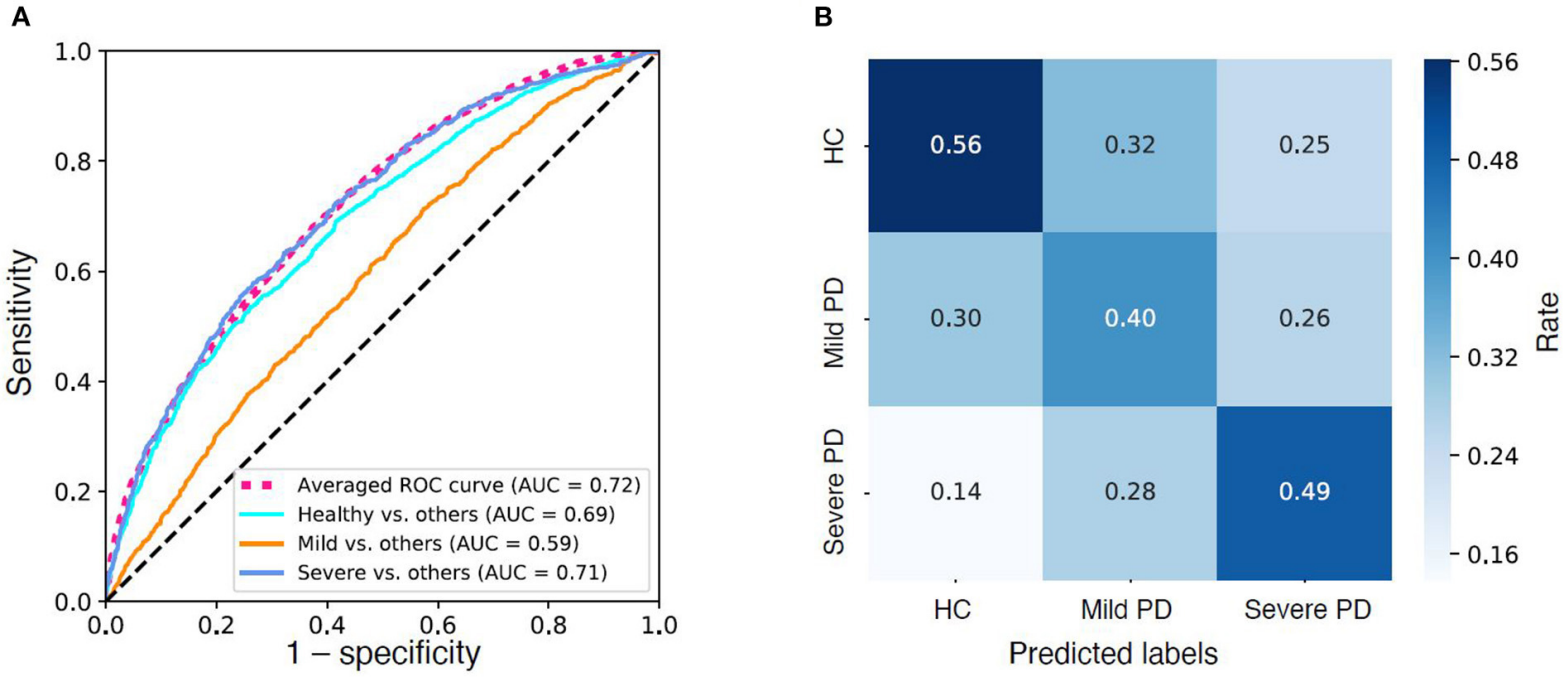
Model performance. **(A)** Receiver operator characteristic (ROC) curves of the three “one-*vs*.-rest” base predictive models and the average ROC curve of the combined multi-label predictive model. **(B)** Confusion Matrix of the predictive model. Color density indicates rate.

EDR (axiUm^®^, Exan software, Las Vegas, Nevada, USA) data of patients who received at least one COE at TUKSoD between January 1, 2017 and August 31, 2021, was used (*n* = 27,138 patients). Patient exclusion criteria include (1) patients without PD assessment information provided by clinicians and (2) patients with a missing rate of over 65% of candidate predictors of interest (Figure 2). After handling the missing values and diagnoses, the final dataset consisted of 18,553 unique patients (see Table 1). Patients with PD diagnoses were grouped into three categories: (1) healthy control (HC), (2) mild PD, and (3) severe PD (see Table 2). These patient data were split into 80% training (*n* = 14,842) and 20% testing (*n* = 3,711) sets. The training set was used for model training, and the testing set was used for model evaluation.

**Table 1:** Characteristics of the studied cohort.

**Characteristics**	**Healthy control**	**Mild PD**	**Severe PD**	**All patients**
Number of patients	8,973	6,108	3,472	18,553
Demographics				
**Age, N(%)**				
15–34	2,816 (31.4)	1,677 (27.5)	919 (26.5)	5,412 (29.2)
35–54	2,655 (29.6)	1,889 (30.9)	1,019 (29.3)	5,563 (30.0)
55–74	2,923 (32.6)	2,123 (34.8)	1,292 (37.2)	6,338 (34.2)
>75	579 (6.5)	419 (6.9)	242 (7.0)	1,240 (6.7)
Sex female, N(%)	5,259 (58.6)	3,556 (58.2)	1,939 (55.8)	10,754 (58.0)
**Race, N(%)**				
White	1,101 (12.3)	735 (12.0)	362 (10.4)	2,198 (11.8)
Black	2,621 (29.2)	1,787 (29.3)	986 (28.4)	5,394 (29.1)
Asian	150 (1.7)	112 (1.8)	61 (1.8)	323 (1.7)
Native	11 (0.1)	6 (0.1)	2 (0.1)	19 (0.1)
Multiracial	38 (0.4)	89 (1.5)	9 (0.3)	136 (0.7)
**Comorbidities, N(%)**				
Cardiovascular	3,419 (38.1)	2,327 (38.1)	1,285 (37.0)	7,031 (37.9)
Nephrology	2,892 (32.2)	2,006 (32.8)	1,082 (31.2)	5,980 (32.2)
Neurology	170 (1.9)	118 (1.9)	78 (2.2)	366 (2.0)
Hematology	264 (2.9)	157 (2.6)	106 (3.1)	527 (2.8)
Endocrinology	20 (0.2)	7 (0.1)	1 (0.0)	28 (0.2)
Rheumatology	116 (1.3)	72 (1.2)	42 (1.2)	230 (1.2)
Pulmonary	49 (0.5)	46 (0.8)	17 (0.5)	112 (0.6)
Gastroenterology	125 (1.4)	77 (1.3)	44 (1.3)	246 (1.3)
Infectious diseases	85 (0.9)	54 (0.9)	25 (0.7)	164 (0.9)
Cancer	40 (0.4)	36 (0.6)	18 (0.5)	94 (0.5)
Osteoporosis	44 (0.5)	29 (0.5)	13 (0.4)	86 (0.5)
**Caries risk assessment, N(%)**				
Low	1,143 (12.7)	397 (6.5)	378 (10.9)	1,918 (10.3)
Moderate	3,417 (38.1)	1,918 (31.4)	874 (25.2)	6,209 (33.5)
High	3,350 (37.3)	2,807 (46.0)	1,507 (43.4)	7,664 (41.3)
Radiographs taken, N(%)	1,476 (16.4)	836 (13.7)	414 (11.9)	2,726 (14.7)
Tobacco use	1,073 (12.0)	1,340 (21.9)	950 (27.4)	3,363 (18.1)
Alcohol use	1,743 (19.4)	1,130 (18.5)	719 (20.7)	3,592 (19.4)
Recreational drug use	960 (10.7)	691 (11.3)	387 (11.1)	2,038 (11.0)
Pregnant	97 (1.1)	31 (0.5)	5 (0.1)	133 (0.7)
Number of teeth, Median [IQR]	27.0[22.0, 28.0]	25.0[20.0, 28.0]	22.0[14.0, 27.0]	26.0[20.0, 28.0]

IQR, Interquartile range.

**Table 2:** Class label definition.

**Class to predict**	**Clinical diagnosis**
Healthy control (HC)	• Healthy periodontium
	• Healthy periodontium with attachment loss
	• Gingivitis
Mild PD	• Slight perio (Stage 1) Localized
	• Slight perio (Stage 1) Generalized
	• Moderate perio (Stage 2) Localized
	• Moderate perio (Stage 2) Generalized
Severe PD	• Severe perio (Stage 3/4) Localized
	• Severe perio (Stage 3/4) Generalized
	• Aggressive periodontitis—Localized
	• Aggressive periodontitis—Generalized

PD, Periodontal disease.

### Developing and testing automated computer applications to phenotype PD diagnoses and medical history information

As EDR is intended to support patient care and not research, patients' clinical information may not be readily available in an analyzable format. For example, patients' PD diagnoses may not be reported using diagnosis codes, as dentists get reimbursed through procedures and not diagnoses. Therefore, two computational approaches were developed that automatically provide PD diagnoses from periodontal charting data and clinical notes per the 2017 American Academy of Periodontology (AAP) classifications. Similarly, patients' medical histories and behavior factor information were extracted from free-text format using natural language processing (NLP) algorithms. Details on developing and testing these computer applications are described elsewhere (Patel et al., 2022).

### Missing value imputation

One of the challenges with using EDR data for research is the missing data, as the EDR data has not been collected for research. Missingness of variables can be found in Table 3. Variables with high missing values were excluded as candidate predictors, and the missing values were imputed according to value types. Specifically, missing values of categorical candidate predictors, such as medical histories, were imputed using the most frequent values. While for the continuous candidate predictors, such as age and teeth number, missing values were imputed using the median values to avoid skewed distribution of the imputed data. Of note, to avoid information leaking, missing values of the testing set were imputed using imputation values in the training set.

**Table 3:** Missingness of the studied variables.

**Variable**	**Missing number**	**Missing rate**
Age	0	0.00
Sex	0	0.00
Race	10,631	0.57
Ethnicity	17,394	0.93
Language	3,301	0.18
Insurance	0	0.00
Comorbidity: CARDIOLOGY category	615	0.03
Comorbidity: NEPHROLOGY category	676	0.04
Comorbidity: NEUROLOGY category	677	0.04
Comorbidity: HEMATOLOGY / ONCOLOGY category	676	0.04
Comorbidity: ENDOCRINOLOGY category	676	0.04
Comorbidity: RHEUMATOLOGY category	676	0.04
Comorbidity: PULMONARY category	1,038	0.06
Comorbidity: GASTROENTEROLOGY category	676	0.04
Comorbidity: INFECTIOUS DISEASES category	676	0.04
Comorbidity: Cancer CATEGORY	676	0.04
Comorbidity: Bisphosponates/Osteoporosis	676	0.04
Comorbidity: Other	676	0.04
Caries risk assessment at visit	2,804	0.15
Radiographs taken	0	0.00
Tobacco use, currently using	484	0.03
Alcohol	1,550	0.08
Recreational drugs, currently using	485	0.03
Preg999t	6,629	0.35
ASA classification	0	0.00
DMFT	0	0.00
DMFS	0	0.00
Number of teeth present	0	0.00
Plaque index: % of sites with plaque	12,371	0.66
Bleeding on probing: % of sites bleeding	10,684	0.57
Bone loss	11,292	0.60
Boneloss level in UR Quad	11,655	0.62
Boneloss level in UR Quad	11,670	0.62
Boneloss level in UR Quad	11,662	0.62
Boneloss level in UR Quad	11,653	0.62
Maximum boneloss % in any quad	5,352	0.29
Subgingival calculus upper right quadrant	11,534	0.62
Subgingival calculus lower right quadrant	11,534	0.62
Subgingival calculus upper left quadrant	11,534	0.62
Subgingival calculus lower left quadrant	11,534	0.62
General risk factor: Inadequate home plaque control	6,205	0.33
General risk factor: Inadequate pt compliance	12,437	0.67
General risk factor: Smoking habit	12,437	0.67
General risk factor: High level of stress	12,437	0.67
General risk factor: Bruxism/parafunctional habit	12,437	0.67
General risk factor: Diabetes mellitus	12,437	0.67
General risk factor: Other systemic medical condition	12,437	0.67
General risk factor: Medications affecting periodontium	12,437	0.67
Local risk factor: Perio attachment loss	12,437	0.67
Local risk factor: Tooth mobility	12,437	0.67
Local risk factor: Furcation involvement	12,437	0.67
Local risk factor: High caries activity	12,437	0.67
Local risk factor: Defective restorations	12,437	0.67
Local risk factor: Removable partial denture(s)	12,437	0.67
Local risk factor: Tooth crowding/root proximity/open contacts	12,437	0.67
Local risk factor: Abnormal tooth anatomy	12,437	0.67
Local risk factor: Radiographic findings	12,437	0.67
Local risk factor: Increased probing depths	12,437	0.67
Self-reported dental health condition	3,940	0.21
Pain level	3,872	0.21
Chewing	4,049	0.22
Speaking trouble	3,957	0.21
Self Image, uneasy or troubled	4,168	0.22
Anxiety, yesterday	3,948	0.21
Anxiety, today	4,046	0.22
Clench or grind	4,107	0.22
Gum trouble	4,122	0.22
Brush frequency	4,092	0.22
Floss frequency	4,162	0.22
Caries risk assessment at visit	4,761	0.25
Dental history: Scaling/surgery for perio disease	7,989	0.43
Dental history: Oral surgery	7,989	0.43
Dental history: injury/trauma to teeth, jaw or gums	7,989	0.43
Dental history: Oral cancer	7,989	0.43
Dental history: Jaw muscle or joint pain (TMJ disorder)	7,989	0.43
Dental history: Orthodontic tooth movement	7,989	0.43

ASA, American Society of Anesthesiologists; DMFT, Calculation of Decayed, Missing, Filled index for number of teeth; DMFS, Calculation of Decayed, Missing, Filled index for number of teeth.

### Model training

The predictive model was based on the XGBoost algorithm (Chen and Guestrin, 2016). XGBoost is an advanced powerful ML model, which combines multiple decision trees to construct a strong model. XGboost has been used in health data analysis tasks and achieved remarkable performances (Khera et al., 2021; Pieszko and Slomka, 2021; Fang et al., 2022) As this is a multi-class classification task (having three classes, i.e., healthy control, mild PD, and severe PD to predict), a one-vs.-all strategy was used. Specifically, the predictive model was the composition of three base models, one predicting HCs from all PDs, one predicting mild PDs from HCs and severe PDs, and one predicting severe PDs from HCs and mild PDs. The rationale of classifying patients into healthy control, mild, and severe is to group the categories on a higher level. For example, our automated phenotyping algorithm (Patel et al., 2022) automatically classifies patients into healthy periodontium, healthy but reduced periodontium, gingivitis classes, and Stage I–Stage IV periodontitis classes with Grade A–C. Our automated algorithm also determined the extent of periodontitis, such as localized and generalized. This includes a total of 24 distinct PD diagnosis categories, as suggested by the 2017 AAP classification system. However, for ML to distinguish all these 24 categories and predict the risk would provide very low accuracy because of “too much variability” in the risk profiles. Moreover, most importantly, clinically, it may not be very relevant at the chairside to predict the risk in one of the 24 categories because the close resembles many of the categories and their treatment planning. For instance, localized gingivitis and generalized gingivitis treatments would be fairly similar, so those categories could be consolidated into one category to reduce “variability” for the ML model to provide optimal performance.

The predictive model was trained using the training set of 18,553 patients (see Figures 1, 2). In order to determine the optimal hyperparameters of the XGBoost, a five-fold cross-validation strategy was used (see Figure 2). Specifically, the training set was split into five-folds with equal size. Each time, a fold was used as the hold-out validation set to calculate model performance, while the remaining four-folds were used to train the model. The procedure was repeated five times to make sure each fold was used as the hold-out validation set once. The optimal hyperparameters can be determined by the best averaged prediction performance in the five-fold validation sets within the training set. Table 4 lists the optimal hyperparameters we achieved after model training. After that, the model was retrained using the whole training set (see Figure 2).

**Table 4:** Optimal hyperparameters of the trained predictive model (XGBoost).

• Maximum depth of a tree: 5 (Increasing this value will make the model more complex and more likely to overfit)
• Learning rate: 0.05 (Step size shrinkage used in update to prevents overfitting. After each boosting step, we can directly get the weights of new features, and eta shrinks the feature weights to make the boosting process more conservative).
• Lambda: 10 (Weight of L2 regularization term. Increasing this value will make model more conservative).
• Minimum sum of instance weight (hessian) needed in a child: 0.1
• Class weight: “balanced” (Balancing the weights of classes, according to sample sizes).

### Model evaluation

Model evaluation was performed on the testing set. Prediction performance was measured by the area under the receiver operating characteristics curve (AUC) and the confusion matrix to compute prediction accuracy, precision, recall, and F-1 measure. The training and testing sets were randomly split by the model; therefore, the cohort statistics of the training and testing data sets are not different.

### Predictor contribution interpretation

To enhance model interpretability, the SHapley Additive exPlanation (SHAP) was utilized, a well-designed tool which is able to interpret output (e.g., decision) of any ML models (Lundberg et al., 2017). The values of each predictor to assess their contributions in distinguishing each class from the others was calculated.

## Results

### Patient demographics and medical history of dental patients

Our sample consisted of 27,138 unique dental patients who received at least one COE between January 1, 2017 and August 31, 2021. Our patients' most common age group was 58–67 years (19%), followed by 48–57 years (18%). African American was the most frequent race (28%), followed by Whites (12%). The majority of our patients were females (57%). Thirty-seven (*n* = 10,148) percent of our dental patients had at least one cardiovascular disease condition. Five percent (*n* = 1,476) of our patients had at least one Nephrology condition, 19% (*n* = 5,135) had at least one Neurology condition, 9% (*n* = 2,400) had Hematology and Oncology conditions, and 18% (*n* = 4,973) had Rheumatology conditions (see Table 1). This summary report was generated before excluding patients with extremely high missing data.

### Social and behavioral habits, DMFT, DMFS, teeth and plaque index scores

Nineteen percent (*n* = 5,022) of our patients smoked cigarettes and 18% (*n* = 4,979) drank alcohol. Eleven percent (*n* = 2,901) of patients use at least one recreational drug, such as cocaine, marijuana, or methamphetamine. The mean Decay Missing Filled Teeth (DMFT) index was 6.06. The mean Decay Missing Filled Surface (DMFS) index was 16.74. Our patients had an average of 22 teeth (presence of teeth) (SD = 8.82, CI = 0.10). The average plaque index score was 73.36. It was discovered that 23% (*n* = 6,357) of patients do not comply with regular brushing, flossing, and use of the mouth wash. Twenty percent (*n* = 5,417) of patients mentioned inadequate home plaque control, 16% (*n* = 4,272) had teeth mobility, and 10% (*n* = 2,639) had defective restoration. Six percent (*n* = 1,618) of our patients reported having high-stress levels, 5% (*n* = 1,381) had bruxism, 4% (*n* = 1,120) took medications that affected their periodontium, and 3% (*n* = 866) had tooth crowding (see Table 1).

### Performance of the prediction model

**Figure 3:**
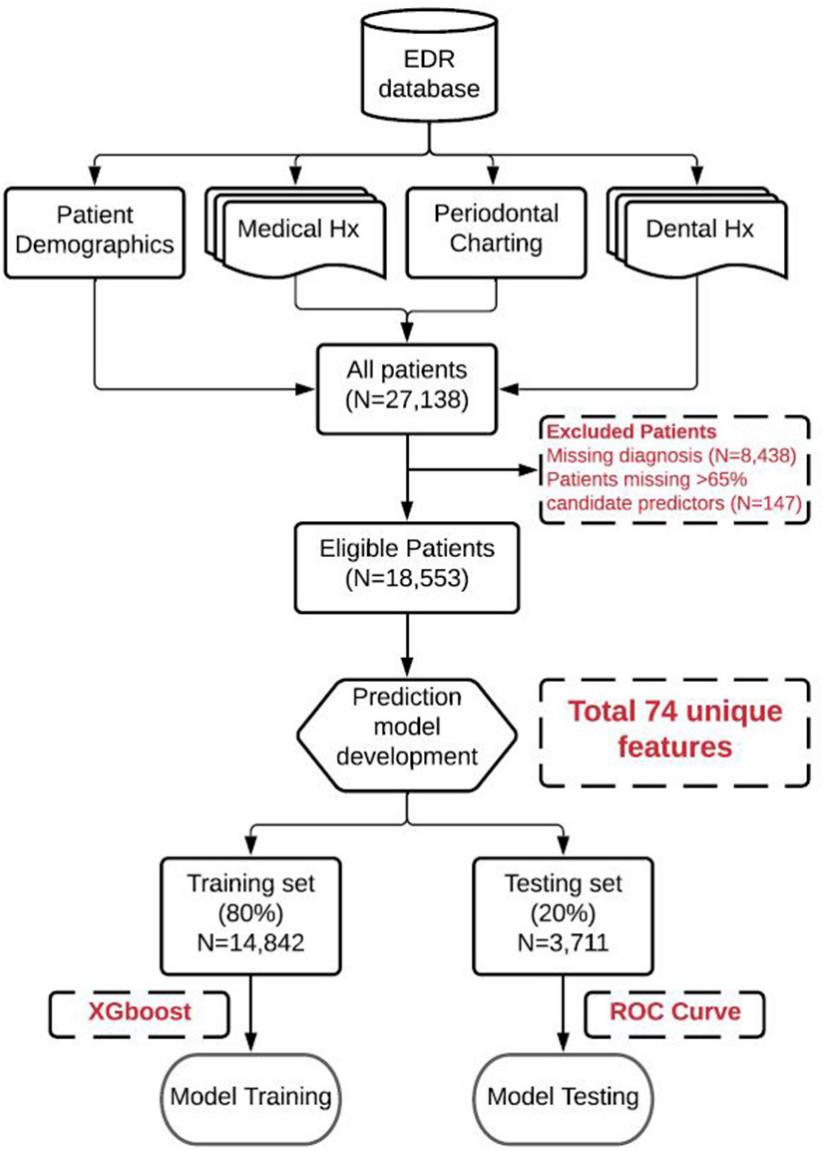
Data extraction, cleaning, preprocessing, and machine learning model training and teasing (Overall workflow).

As demonstrated in the Methods, section, the prediction model was built on the final dataset of 18,553 patients (after excluding patients with high missing data). We achieved an AUC of 0.72 (weighted average of three base models) in distinguishing HC, mild PD, and severe PD from each other (see Figure 3; Table 5). When looking into the “one-*vs*.-rest” base models, the models work well in distinguishing HCs from PDs (AUC = 0.69, F1-score = 0.66) as well as in distinguishing the severe PDs from HCs and mild PDs (AUC = 0.71, F1-score = 0.30) (see Table 5).

**Table 5:** Prediction performance of the model.

	**AUC**	**Precision**	**Recall**	**F1-score**
**Healthy vs. others (PD)**	0.69	0.56	0.81	0.66
**Mild vs. others**	0.59	0.40	0.28	0.33
**Severe vs. others**	0.71	0.49	0.22	0.30
**Combined model**	0.72	0.501	0.521	0.481

AUC, Area under the receiver operating characteristic curve; PD, Periodontal disease.

Precision, Recall, and F1-score of the combined model were calculated as weighted average of the three base models.

### Risk factors and features identified using a data-driven approach

**Figure 4:**
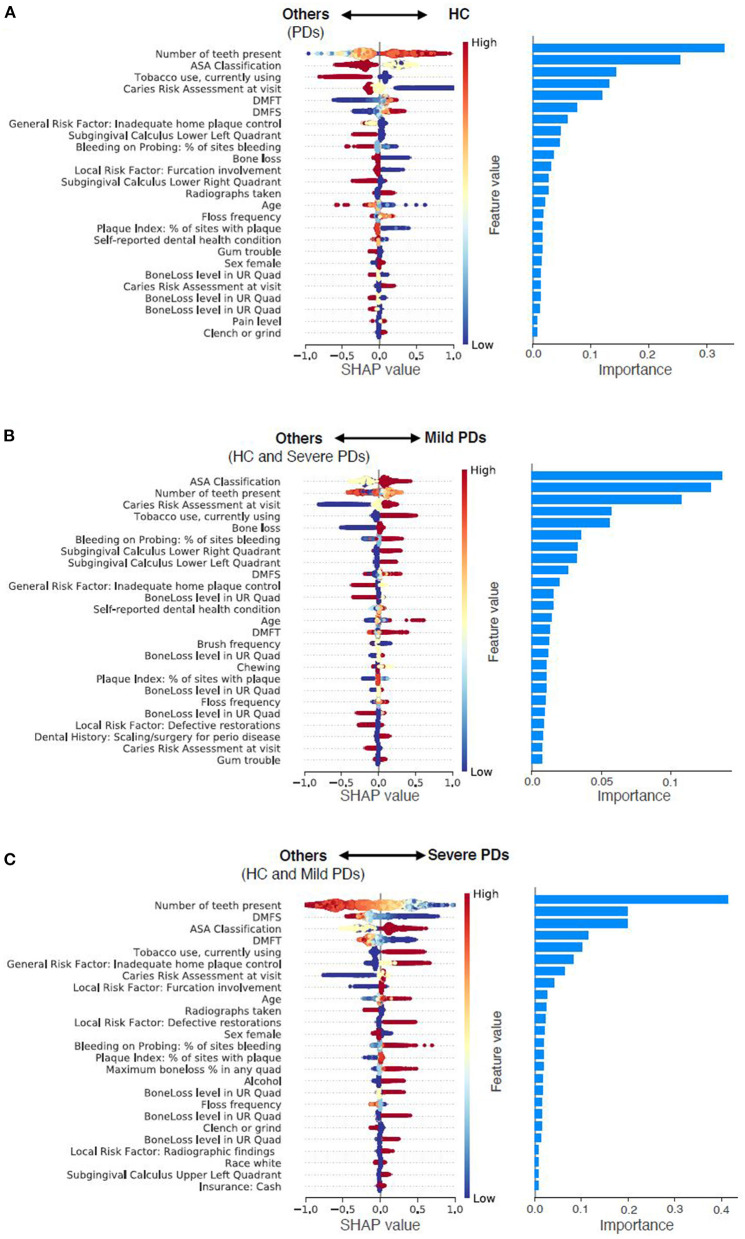
Predictor contributions. Contributions of top 25 predictors in predicting HC from PDs **(A)**, predicting Mild PD from others (HC and Severe PD) **(B)**, and predicting Severe PD from others (HC and Mild PD) **(C)**. For the left column, each dot indicates a sample, color density of the dot indicates normalized value of the specific feature of the specific sample, and horizontal axis indicates SHAP values. High positive SHAP value indicates that the specific feature value has a high positive contribution on prediction, and vice versa. For instance, smaller values of number of teeth present are strong indicator of Severe PD. For the right column, importance of each predictor was calculated as the mean absolute SHAP value of the predictor.

New associations and novel features that include social determinants of health, medical conditions, patients' oral health habits, and patients' overall health (see Figure 4; Table 6). We categorized these risk factors into modifiable and non-modifiable risk factors. The rationale for categorizing these risk factors into these two groups is that clinicians would only be interested in knowing the modifiable risk factors to take preventive approaches and provide patient education at the chairside. The non-modifiable risk factors include patients' age, gender, race, insurance status, pregnancy, number of teeth present, periodontal bone loss, periodontal attachment loss, and furcation involvement. For example, older patients are more likely to suffer from periodontitis than younger individuals. Similarly, the Black race and patients without dental insurance are more likely to suffer from periodontitis. Some of the periodontal findings, such as bone loss and furcation involvement were purposefully categorized in the non-modifiable risk factors category because even though we used these factors toward prediction, the condition is irreversible, and therefore, clinicians have no control over these factors.

**Table 6:** Details of predictors of each base one-*vs*.-rest model.

**Healthy vs. others (PD)**		**Mild vs. others**		**Severe vs. others**	
**Feature name**	**Importance**	**Feature name**	**Importance**	**Feature name**	**Importance**
Number of teeth present	0.329337507	ASA classification	0.136346847	Number of teeth present	0.413637727
ASA classification	0.254108638	Number of teeth present	0.128605053	DMFS	0.198916376
Tobacco use, currently using	0.142421514	Caries risk assessment at visit	0.107289292	ASA classification	0.197939038
Caries risk assessment at visit	0.131898284	Tobacco use, currently using	0.057154436	DMFT	0.115095645
DMFT	0.119128674	Bone loss	0.055876885	Tobacco use, currently using	0.101688631
DMFS	0.076166414	Bleeding on Probing: % of sites bleeding	0.035499241	General risk factor: Inadequate home plaque control	0.082106486
General risk factor: Inadequate home plaque control	0.060233109	Subgingival calculus lower right quadrant	0.032867499	Caries risk assessment at visit	0.063146502
Subgingival calculus lower left quadrant	0.048033334	Subgingival calculus lower left quadrant	0.032012567	Local risk factor: Furcation involvement	0.040414538
Bleeding on Probing: % of sites bleeding	0.045954991	DMFS	0.025991267	Age	0.025732813
Bone loss	0.035547402	General risk factor: Inadequate home plaque control	0.020037225	Radiographs taken	0.024807494
Local risk factor: furcation involvement	0.030682651	BoneLoss level in UR Quad	0.015393356	Local risk factor: Defective restorations	0.022771299
Subgingival calculus lower right quadrant	0.026781825	Self-reported dental health condition	0.015326378	Sex female	0.020993775
Radiographs taken	0.026514396	Age	0.014268779	Bleeding on Probing: % of sites bleeding	0.01914837
Age	0.021277493	DMFT	0.013263618	Plaque Index: % of sites with plaque	0.018625483
Floss frequency	0.017227829	Brush frequency	0.012612002	Maximum boneloss % in any quad	0.017772963
Plaque Index: % of sites with plaque	0.01679814	BoneLoss level in UR Quad	0.011913223	Alcohol	0.017202327
Self-reported dental health condition	0.01649419	Chewing	0.010722518	BoneLoss level in UR Quad	0.017198784
Gum trouble	0.016416514	Plaque index: % of sites with plaque	0.010572524	Floss frequency	0.015760772
Sex female	0.015495853	BoneLoss level in UR Quad	0.010420221	BoneLoss level in UR Quad	0.01545079
BoneLoss level in UR Quad	0.013729406	Floss frequency	0.00962163	Clench or grind	0.015447081
Caries risk assessment at visit	0.012893611	BoneLoss level in UR Quad	0.009416237	BoneLoss level in UR Quad	0.012436057
BoneLoss level in UR Quad	0.012722277	Local risk factor: Defective restorations	0.008430392	Local Risk Factor: Radiographic findings	0.007904406
BoneLoss level in UR Quad	0.011671119	Dental history: Scaling/surgery for perio disease	0.007853939	Race white	0.007582215
Pain level	0.008060957	Caries risk assessment at visit	0.007725936	Subgingival calculus upper left quadrant	0.007331495
Clench or grind	0.007378913	Gum trouble	0.007650368	Insurance: Cash	0.007083471
Brush frequency	0.006935218	General Risk Factor: Inadequate pt compliance	0.007123779	Insurance: Medicaid	0.006893774
Comorbidity: CARDIOLOGY category	0.006225374	Radiographs taken	0.00657481	Self-reported dental health condition	0.006705926
Maximum boneloss % in any quad	0.005455129	General risk factor: Diabetes mellitus	0.006210369	Anxiety, today	0.006574302
Dental history: Scaling/surgery for perio disease	0.005196851	Alcohol	0.00562494	Dental history: Scaling/surgery for perio disease	0.006312543
Local risk factor: High caries activity	0.004980592	Local risk factor: Tooth mobility	0.004714876	Race black	0.006310361
Anxiety, yesterday	0.004811309	Self Image, uneasy or troubled	0.004550776	Brush frequency	0.006111717
Insurance: Cash	0.004598638	Maximum boneloss % in any quad	0.004517836	Local risk factor: Increased probing depths	0.005483611
Speaking trouble	0.0045798	Dental history: Jaw muscle or joint pain (TMJ disorder)	0.004295081	BoneLoss level in UR Quad	0.005455468
Preg	0.004357389	Local risk factor: Furcation involvement	0.004074463	Anxiety, yesterday	0.005027993
Local risk factor: Tooth crowding/root proximity/open contacts	0.003708631	Comorbidity: CARDIOLOGY category	0.004020691	Dental history: injury/trauma to teeth, jaw or gums	0.004409812
Alcohol	0.003623726	Insurance: Medicaid	0.003989982	Speaking trouble	0.003840377
Subgingival calculus upper right quadrant	0.003599183	Anxiety, yesterday	0.003616055	Caries risk assessment at visit	0.003786845
Subgingival calculus upper left quadrant	0.002787969	Comorbidity: HEMATOLOGY / ONCOLOGY category	0.003587578	General risk factor: High level of stress	0.003654674
Local risk factor: Tooth mobility	0.002725839	Clench or grind	0.003407459	Chewing	0.003567741
BoneLoss level in UR Quad	0.002553322	Sex female	0.002990388	Comorbidity: NEPHROLOGY category	0.003430167
Comorbidity: NEPHROLOGY category	0.00250311		0.002985413	Preg	0.003143014
Race black	0.002485255	Local risk factor: Radiographic findings	0.002627898	Self Image, uneasy or troubled	0.003137945
General risk factor: Smoking habit	0.002428178	Subgingival calculus upper right quadrant	0.002615314	Local Risk factor: Removable partial denture(s)	0.002948748
Insurance: Medicaid	0.002332768	Subgingival calculus upper left quadrant	0.002573571	Comorbidity: CARDIOLOGY category	0.002377946
Anxiety, today	0.00233276	General risk factor: Smoking habit	0.002408407	Local risk factor: High caries activity	0.001959847
Local risk factor: Defective restorations	0.002282311	Speaking trouble	0.002381834	Recreational drugs, currently using	0.001717853
Self Image, uneasy or troubled	0.002042823	Dental history: Oral surgery	0.001985606	Subgingival calculus lower right quadrant	0.001610996
General risk factor: Diabetes mellitus	0.001805591	Pain level	0.001944372	General risk factor: Bruxism/parafunctional habit	0.001568569
General risk factor: High level of stress	0.00171883	Local risk factor: High caries activity	0.001911726	General risk factor: Diabetes mellitus	0.001561241
General risk factor: Inadequate pt compliance	0.001595587	Insurance: Cash	0.001448046	Dental history: Oral surgery	0.001384793
Local risk factor: Increased probing depths	0.001348896	Recreational drugs, currently using	0.001429585	Gum trouble	0.001245809
Dental history: Orthodontic tooth movement	0.001155873	Race white	0.001364392	Subgingival calculus upper Right Quadrant	0.001141013
Insurance: Private	0.00106112	Race black	0.001164276	Dental history: Jaw muscle or joint pain (TMJ disorder)	0.001038408
Local risk factor: Removable partial denture(s)	0.001058387	Insurance: Private	0.001053769	Pain level	0.000929357
Local risk factor: Abnormal tooth anatomy	0.001027395	Local risk factor: Removable partial denture(s)	0.00095389	Bone loss	0.000922859
Local risk factor: Radiographic findings	0.000971377	General risk factor: High level of stress	0.000845793	Local Risk Factor: Abnormal tooth anatomy	0.000921286
Dental history: Oral surgery	0.000918839	Language: English	0.000838721	Insurance: Private	0.000725477
Race white	0.000809216	Anxiety, today	0.000767338	Local risk factor: Perio attachment loss	0.000721364
Recreational drugs, currently using	0.000765298	General Risk Factor: Medications affecting periodontium	0.000734203	Subgingival calculus lower left quadrant	0.000597729
General risk factor: Bruxism/parafunctional habit	0.000698673	Local risk factor: Tooth crowding/root proximity/open contacts	0.000714158	Comorbidity: HEMATOLOGY / ONCOLOGY category	0.000522715
Chewing	0.000625421	General risk factor: Other systemic medical condition	0.000588053	Local risk factor: Tooth mobility	0.000501615
Language: English	0.000196944	Race asian	0.000489697	General risk factor: Medications affecting periodontium	0.000480412
Race asian	0.000172019	Comorbidity: NEPHROLOGY category	0.000312514	Comorbidity: Other	0.000408197
Comorbidity: HEMATOLOGY / ONCOLOGY category	0.000154656	Local risk factor: Abnormal tooth anatomy	0.000277071	Race asian	0.000398896
General risk factor: Medications affecting periodontium	0.000125101	General risk factor: Bruxism/parafunctional habit	0.00019122	Comorbidity: NEUROLOGY category	0.000338069
Race multiracial	9.70E-05	Comorbidity: PULMONARY category	0.000168363	General risk factor: Inadequate pt compliance	0.000308447
Comorbidity: RHEUMATOLOGY category	5.27E-05	Dental history: Orthodontic tooth movement	0.00016245	General risk factor: Smoking habit	0.000269852
Dental history: Jaw muscle or joint pain (TMJ disorder)	4.39E-05	Dental history: injury/trauma to teeth, jaw or gums	0.000134683	Dental history: Orthodontic tooth movement	0.000179533
General risk factor: Other systemic medical condition	3.23E-05	Comorbidity: RHEUMATOLOGY category	0.000134487	Insurance: Ryan White	0.000141657
Dental history: injury/trauma to teeth, jaw or gums	3.18E-05	Comorbidity: GASTROENTEROLOGY category	0.00011029	Comorbidity: Bisphosponates/Osteoporosis	0.000135203
Insurance: Ryan White	2.07E-05	Local risk factor: Perio attachment loss	0.000105196	Language: English	9.20E-05
Comorbidity: PULMONARY category	1.72E-05	Insurance: Ryan White	9.09E-05		7.77E-05
Comorbidity: NEUROLOGY category	1.29E-05	Local risk factor: Increased probing depths	8.91E-05	Local risk factor: Tooth crowding/root proximity/open contacts	5.39E-05
Comorbidity: Other	1.21E-05	Comorbidity: NEUROLOGY category	8.14E-05	Comorbidity: Cancer CATEGORY	4.48E-05
Comorbidity: Bisphosponates/Osteoporosis	8.86E-06	Comorbidity: Cancer CATEGORY	2.66E-05	Race multiracial	1.89E-05

The modifiable risk factors were further classified into four subcategories: (1) Social determinants of health, (2) Social habits, (3) Oral health habits, and (4) Systemic diseases. Patients that belonged to the following groups had a higher incidence of periodontitis than the HC group. Patients who lived at a farther distance from the dental school had higher risk of PD. Similarly, smokers, who drank alcohol, patients who aren't compliant with brushing and flossing also had higher incidence of PD. Patients with certain dental conditions such as crowded teeth, teeth grinding, dental anxiety, speaking trouble, self-image issues, defective restorations, abnormal tooth anatomy, presence of removable partial dentures, higher DMFT, and DMFS index, a higher number of carious teeth, past history of periodontal treatments had higher rates of PD. Finally, patients who had multiple systemic conditions were also at advanced risk of PD.

## Discussion

This study demonstrated the successful creation of a prediction of PD with moderate to high accuracy (72% average AUC). This is the first study that used 74 features to develop and test PD prediction models. Novel features, including social determinants of health, social habits, oral health habits, and systemic diseases were discovered. This new information would provide clinicians with identifying high-risk PD patients to take preventive approaches for disease prevention with the hope of reducing disease prevalence, reducing healthcare costs, and improving quality of life.

A few studies in dentistry attempted to develop prediction models for PD using the EDR data. Shimpi et al. (2019) developed a prediction model that could be applicable at point-of-care using supervised ML methods. The author compared the performances of ML models and found that Decision Tree and Artificial Neural Network provided higher accuracy (87%) in classifying patients with high or low PD risks. Similarly, Thyvalikakath Thankam et al. (2015) and Hegde et al. (2019) have utilized EDR data to predict the risk of periodontitis. The accuracy with automated data was 92%, the recall was 73%, and precision was 93%. Compared to these studies, our model performed slightly inferior [precision (0.50), recall (0.53)]; however, these studies were restricted by the number of risk factors. We studied a wider range of variables in EDR that were collected in daily patient care. Moreover, instead of predicting PDs and HCs, this study proposed to predict HCs, mild PDs, and severe PDs, which made our task more difficult but more useful for the clinicians. In this study, the data decided (unlike expert driven models) which risk factors have a stronger influence on the PD than the traditional risk factors; especially, there is a significant shift in the prevalence of risk factors.

As demonstrated in section “Risk factors and features identified using a data-driven approach,” new features and modifiable risk factors were discovered that could drive the periodontitis risk. For example, patients who reported using recreational drugs, smoking, and alcohol are at high risk of PD, so dental clinicians can provide counseling programs to these patients. Many tobacco cessation programs are available for patients who smoke tobacco, which could be referred by dental clinicians (Myers Virtue et al., 2017). Next, it was discovered that patients who live farther away had a higher rate of periodontitis, which could be due to the lack of available, affordable dental care in remote areas. Most of our patients come from under represented and lower socioeconomic areas, so they may not afford to go to private dental clinics for their treatments. This information would help us determine the strategies for zip code locations to conduct dental camps and outreach dental programs to help these patients get regular dental and preventive treatments. Next, patients' oral health factors include inadequate brushing and flossing, crowded teeth, teeth grinding, dental anxiety, and defective restorations. Patients with defective restorations should be replaced as early as possible upon identification, and the teeth grinding can be managed by providing mouth guards to the patients. Dental anxiety and other mental conditions can be managed by providing patient counseling or referring patients to specialists. In summary, our approach would allow dental clinicians to provide more holistic care than just dental care. It is also important to note that some risk factors, such as the presence of removable partial dentures (RPDs), could result from high dental caries (the primary reason for losing teeth). It is studied that a higher incidence of dental caries is associated with a higher number of RPDs. However, in this study, we found that patients who had RPDs also had a higher incidence of severe periodontitis. This link could be due to the association between dental caries and periodontitis.

This study encountered some limitations. First, the prediction model was developed only using one institute's dataset; therefore, our study results may not be generalizable. Next, only one ML algorithm was tested, which may have provided superior performance than XGBoost. Behavior factors such as smoking, alcohol, and the use of recreational drugs are self-reported information. Self-reported information may or may not be reliable, which may have an impact on the performance of our prediction model. Only simple imputation methods were utilized to impute missing data. Next, a retrospective study of electronic chart review like this provided information about possible associations between risk factors and PD, however, we did not identify possible causations. To understand the caustic relations, longitudinal studies are warned. In future studies, we will analyze longitudinal EDR data to identify clinical course of periodontitis. We will also test the performance of other more sophisticated algorithms and then test the performance of our prediction model on the external dataset. In addition, a usability study will also be performed with dental clinicians to determine their attitudes, perception, and opinions about the prediction model and its use in real dental clinical settings. Last, a clinical decision support system that can be implemented in EDR to predict the risk of periodontitis at TUKSoD clinics for patient care will be developed. Other data sources such as geographic information systems to identify factors, socioeconomic factors, and criminal history will also be utilized to add more risk factors to this prediction model.

## Conclusion

Our pilot study demonstrated promising results in utilizing the EDR and ML model to predict the risk of PD. This model would provide new information to the clinicians about the PD risks and the factors responsible for the disease progression to take preventive approaches. Further studies are warned to evaluate the performance of our prediction model on the external dataset and determine its usability in clinical settings.

## Data availability statement

The datasets presented in this article are not readily available as they may contain patient identifiable information. In case any researchers want to collaborate and utilize this dataset, a data use agreement and Internal Review Board processes are mandatory. Requests to access the datasets should be directed to JP at patel.jay@temple.edu.

## Ethics statement

The studies involving human participants were reviewed and approved by Temple University Internal Review Board (IRB#28321). The patients/participants provided their written informed consent to participate in this study.

## Author contributions

JP contributed to the conception, design, data acquisition, analysis and interpretation, drafted, critically revised the manuscript, gave full final approval, and agreed to be accountable for all aspects of the work. CS contributed to data analysis and interpretation, revised the manuscript, gave full final approval, and agreed to be accountable for all aspects of the work. MT and JA contributed to conception and interpretation, drafted and critically revised the manuscript, gave full final approval, and agreed to be accountable for all aspects of the work. HW contributed to conception, design, analysis, interpretation, critically revised the manuscript, gave full final approval, and agreed to be accountable for all aspects of the work. RR, VI, and ES student workers helped significantly with data cleaning. All authors contributed to the article and approved the submitted version.

## Funding

This study was funded through start-up funds of the senior author HW.

## Conflict of interest

The authors declare that the research was conducted in the absence of any commercial or financial relationships that could be construed as a potential conflict of interest.

## Publisher's note

All claims expressed in this article are solely those of the authors and do not necessarily represent those of their affiliated organizations, or those of the publisher, the editors and the reviewers. Any product that may be evaluated in this article, or claim that may be made by its manufacturer, is not guaranteed or endorsed by the publisher.
